# NSAIDs, Mitochondria and Calcium Signaling: Special Focus on Aspirin/Salicylates

**DOI:** 10.3390/ph3051594

**Published:** 2010-05-19

**Authors:** Yoshihiro Suzuki, Toshio Inoue, Chisei Ra

**Affiliations:** Division of Molecular Cell Immunology and Allergology, Nihon University Graduate School of Medical Science, Tokyo, Japan

**Keywords:** aspirin, calcium, mitochondria, nonsteroidal anti-inflammatory drug (NSAID), reactive oxygen species

## Abstract

Aspirin (acetylsalicylic acid) is a well-known nonsteroidal anti-inflammatory drug (NSAID) that has long been used as an anti-pyretic and analgesic drug. Recently, much attention has been paid to the chemopreventive and apoptosis-inducing effects of NSAIDs in cancer cells. These effects have been thought to be primarily attributed to the inhibition of cyclooxygenase activity and prostaglandin synthesis. However, recent studies have demonstrated unequivocally that certain NSAIDs, including aspirin and its metabolite salicylic acid, exert their anti-inflammatory and chemopreventive effects independently of cyclooxygenase activity and prostaglandin synthesis inhibition. It is becoming increasingly evident that two potential common targets of NSAIDs are mitochondria and the Ca^2+^ signaling pathway. In this review, we provide an overview of the current knowledge regarding the roles of mitochondria and Ca^2+^ in the apoptosis-inducing effects as well as some side effects of aspirin, salicylates and other NSAIDs, and introducing the emerging role of L-type Ca^2+^ channels, a new Ca^2+^ entry pathway in non-excitable cells that is up-regulated in human cancer cells.

## 1. Introduction

Aspirin (acetylsalicylic acid) is a well-known nonsteroidal anti-inflammatory drug (NSAID) that has long been used as an anti-pyretic and analgesic drug. Other NSAIDs are also generally used to treat pain, inflammation and fever. The anti-inflammatory actions of NSAIDs have been thought to be primarily attributed to inhibition of prostaglandin (PG) synthesis [1]. Aspirin acetylates Ser-530 of cyclooxygenase (COX) I and II, thereby blocking PG and thromboxane A_2_ synthesis, while therapeutic concentrations of aspirin and salicylates inhibit COX II protein expression [2]. However, there is also evidence that certain NSAIDs, including aspirin, salicylates, sulindac, ibuprofen and flurbiprofen have anti-inflammatory and anti-proliferative effects independent of COX activity and PG synthesis inhibition (for a comprehensive review, see [3]). The doses of aspirin used to treat chronic inflammatory diseases are much higher than those required to inhibit PG synthesis. Moreover, salicylate reduces inflammation, although it lacks the acetyl group and is ineffective as a COX inhibitor at therapeutic doses [4,5,6]. In addition, most of these effects have only been observed at high concentrations of the respective NSAIDs, which are 100- to 1000-fold higher than those required to inhibit PG synthesis [3]. Thus, individual NSAID may utilize intrinsic COX-independent mechanisms to exert their anti-inflammatory effects. These effects are mediated through inhibition of certain transcription factors such as nuclear factor-κB (NF-κB), AP-1 and nuclear factor of activated T cells [7,8,9]. Another possible important mechanism of the anti-inflammatory effects may be modulation of the activation of mast cells and basophils, since these cells play pivotal roles in allergic inflammatory reactions. Aspirin has been shown to modulate mast cell degranulation, COX-2 expression and release of pro-inflammatory cytokines [11]. We recently reported that aspirin and salicylates modulate proinflammatory mediator release in mast cells through a COX-independent mechanism in which Ca^2+^ signaling plays a key role [11,12]. Since this issue is close to the main theme of this review, we will discuss it in more detail in the section 2. 

In addition to their anti-inflammatory actions, NSAIDs are emerging as promising antineoplastic drugs. Numerous studies have suggested that the use of NSAIDs, primarily aspirin, decreases the risks of several cancers, including, cancers of the colon and other gastrointestinal organs as well as those of the breast, prostate, lung, ovary and skin [13,14,15,16,17,18,19]. Since PGs inhibit apoptosis and induce the formation of new blood vessels, thereby contributing to tumor growth [20,21,22], COX inhibition may explain a part of the antineoplastic activities of certain NSAIDs. However, NSAIDs have growth inhibitory effects on colon cancer cell lines that do not express the COX-1 and COX-2 enzymes [23,24], and also on mouse embryo fibroblasts that are null for both the COX-1 and COX-2 genes [25]. Such observations are inconsistent with the conventional hypothesis that NSAIDs act primarily or exclusively by inhibiting PG synthesis. NSAIDs have also been shown to induce apoptosis and necrosis in cancer cells (for reviews, see [3,26]), which may be potential mechanisms for their chemopreventive effects. In addition, NSAIDs exhibit multiple effects on a variety of intracellular signaling pathways, including the mitogen-activated protein kinase (MAPK) cascade, ribosomal S6 kinase, signal transducer and activator of transcription 1 and transforming growth factor β. They also modulate several processes, such as cell cycle progression and the activities of nuclear receptor family members, including peroxisome proliferator-activated receptor γ. It remains unclear whether these effects are direct or indirect [3]. These biological effects may also play roles in tumor growth inhibition and/or cell death induction. Thus, the molecular mechanisms underlying the chemopreventive effects of NSAIDs remain a matter of debate. In this review, we will focus on the COX-independent mechanisms of NSAID-induced cell death with special attention to the roles of mitochondria in Section 3. 

Aspirin has various side effects on the gastro-intestinal tract, and primarily causes gastric lesions, ulcerations and erosions [27]. Aspirin also induces immunological side effects, which are collectively referred to as aspirin intolerance (see Section 2). Aspirin intolerance is a disorder that induces urticaria, asthma and anaphylaxis in response to oral administration of the drug [28,29]. Aspirin also potentiates some acute allergies such as food-dependent exercise-induced anaphylaxis (FDEIA), which is a food allergy induced by physical exercise. Recently, aspirin was shown to act as a powerful trigger of anaphylaxis in FDEIA patients [30].

## 2. COX-Independent Modulation of Mast Cell Activation by NSAIDs

Mast cells play critical roles in allergic inflammatory reactions. These cells express the high-affinity IgE receptor (FcεRI) on their cell surface and cross-linking of IgE-bound FcεRI by multivalent antigens induces aggregation of the receptor, which triggers biochemical cascades that lead to cell activation. Upon antigen stimulation, mast cells release various preformed granular substances, such as histamine and serotonin (a process referred as to degranulation), and synthesize and secrete arachidonate (AA) metabolites such as leukotrienes (LTs) and PGs as well as cytokines and chemokines [31]. These chemical mediators cause various pathophysiological events that contribute to acute and chronic inflammation. Therefore, inhibition of the proinflammatory mediator release is a potential mechanism for the anti-inflammatory effects of NSAIDs. Recent studies have revealed that NSAIDs modulate mast cell degranulation, COX-2 expression and release of pro-inflammatory cytokines by affecting heat shock protein and Toll-like receptor-mediated responses [11,32]. In addition, several studies have shown that an atopic background (high levels of serum IgE) is a risk factor for NSAID sensitivity [33]. One key feature of aspirin intolerance is overproduction of cysteinyl LTs (cys-LTs) such as LTC_4_, LTD_4_ and LTE_4_, which are all sequentially synthesized from arachidonic acid. These cys-LTs are potent proinflammatory mediators and cause smooth muscle contraction and increased vascular permeability. Patients with aspirin intolerance have significantly higher levels of cys-LTs in their bronchoalveolar lavage fluid and urine before and after oral aspirin challenge [34]. Moreover, cys-LT synthase activity is predominantly detected in mast cells, which are the major producers of cys-LTs [35,36]. These observations suggest that mast cells may play roles in both the anti-inflammatory effects and side effects of certain NSAIDs, primarily aspirin. To understand the molecular mechanisms underlying aspirin intolerance, we investigated the possible effects of aspirin on cys-LT production in mast cells. Aspirin alone at concentrations ranging from 0.1 to 3 mM had minimal effects on LTC_4_ secretion. However, aspirin had dual effects on antigen-induced LTC_4_ secretion depending on the concentration used. At therapeutic levels (≤0.3 mM), representing the concentrations observed *in vivo* for antipyretic and analgesic use, aspirin enhanced LTC_4_ secretion, while at higher concentrations (>1 mM), it suppressed LTC_4_ secretion [11]. Essentially similar effects were observed with salicylates, which lack inhibitory effects on COX-1 and COX-2 activities [37], thereby indicating that aspirin exerts these effects independently of COX activity. Cytosolic phospholipase A_2_ (cPLA_2_) mediates agonist-induced AA release in most cell types (for reviews, see [38,39]). The catalytic activity of cPLA_2_ is phosphorylation-dependent. Phosphorylation of Ser-505 in cPLA_2_ by extracellular signal-regulated kinase 1/2 (ERK1/2) is necessary for cPLA_2_-mediated AA release following stimulation of various cell types by many different agonists [39,40]. Aspirin stimulates phosphorylation of Ser-505 in cPLA_2_ at concentrations that augment LTC_4_ secretion [11]. Antigen stimulation leads to ERK1/2 activation, as evidenced by increased dual phosphorylation of Thr-202 and Tyr-204, while the MAPK kinase inhibitor U0126 reduces LTC_4_ secretion. These data suggest that ERK1/2 is activated by the upstream kinase MEK1/2, as reported in a variety of cell types [38,39]. Ser-727 in cPLA_2_ is another important site for activation of the enzyme, which is mediated by p38MAPK activated via dual phosphorylation of Thr-180 and Tyr-182 [41]. Unexpectedly, it was found that aspirin at concentrations ranging from 0.1 to 3 mM dose-dependently reduces the activation of ERK1/2 and had no significant effects on the activation of p38MAPK. Collectively, these data indicate that aspirin enhances cPLA_2_ activation independently of the ERK and p38MAPK pathways, thereby suggesting the involvement of another mechanism.

## 3. Modulation of Ca^2+^ Channel Activities by NSAIDs

**Figure 1:**
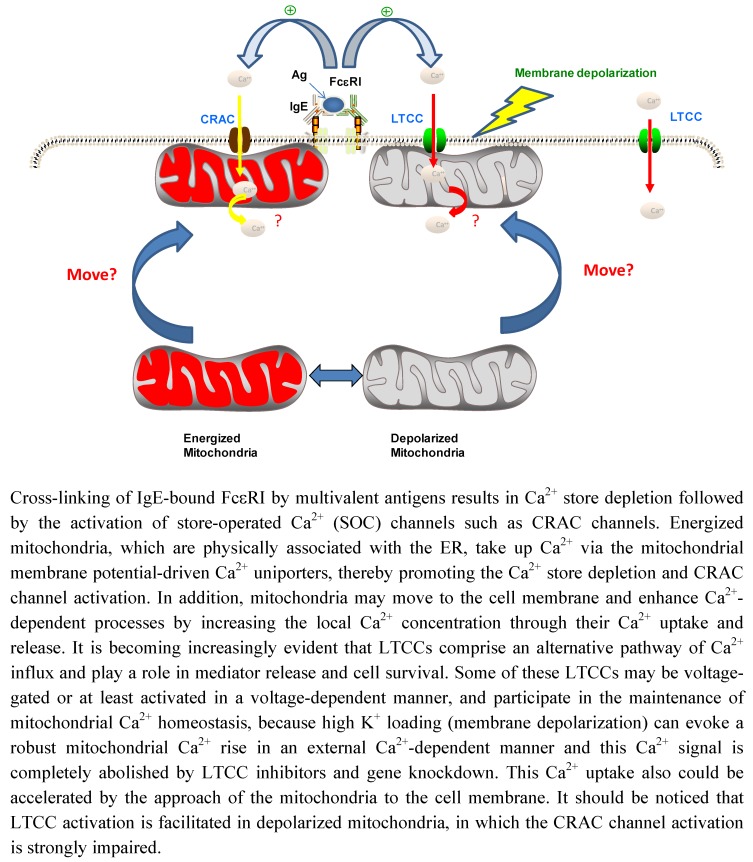
A model for Ca^2+^ signaling in mast cells.

**Figure 2:**
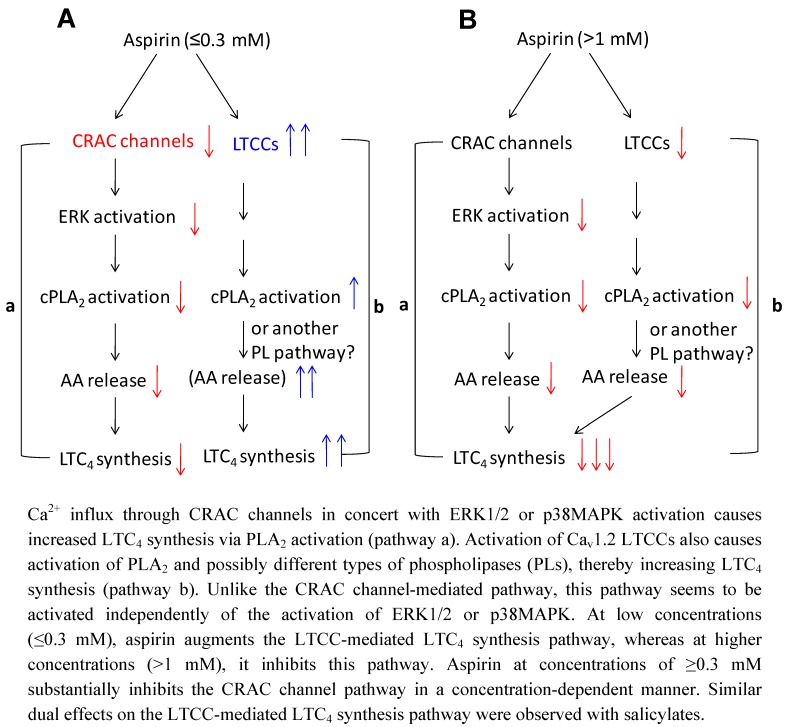
Dual effects of NSAIDs on the novel LTCC-mediated LTC_4_ synthesis pathway.

Ca^2+^ is a highly versatile intracellular second messenger in many cell types, and regulates many complicated cellular processes, including cell activation, proliferation and apoptosis. Elevation of the intracellular Ca^2+^ concentration, mainly through Ca^2+^ entry from the extracellular space, is necessary for the new synthesis and secretion of cys-LTs [31]. Ca^2+^ binds to the amino-terminal C2 domain of cPLA_2_ and leads to its translocation to the nuclear envelope and endoplasmic reticulum (ER) and activation [38,39]. Ca^2+^ is also an important regulator of 5-lipoxygenase, which catalyzes the addition of molecular oxygen to AA. Analyses of Ca^2+^ influx have revealed that aspirin has dual effects on this process depending on the concentration used, similar to the observations for LTC_4_ secretion. Specifically, at low concentrations (≤0.3 mM), aspirin enhanced Ca^2+^ influx, while at high concentrations (>1 mM), it suppressed Ca^2+^ influx [11]. It is widely accepted that store-operated Ca^2+^ entry (SOCE) is the main mode of Ca^2+^ influx in electrically non-excitable cells, including mast cells [42]. SOCE is mediated by store-operated Ca^2+^ (SOC) channels like Ca^2+^ release-activated Ca^2+^ (CRAC) channels, which are activated by depletion of intracellular Ca^2+^ stores. Despite its stimulatory effect on Ca^2+^ influx at low concentrations, aspirin reduces CRAC channel activity. These data suggest that aspirin may stimulate another Ca^2+^ entry pathway. It has long been thought that long-lasting voltage-gated L-type Ca^2+^ channels (LTCCs) represent a characteristic feature of excitable cells. However, pharmacological, molecular and genetic approaches have recently revealed the existence of functional LTCCs or LTCC-like channels in a variety of hematopoietic cells such as B cells, dendritic cells, natural killer cells, neutrophils, mast cells and T cells (for reviews, see [43,44]). Among these, the Ca^2+^ channels in T cells have been the most extensively studied. These cells express a channel (or channels) sharing elements of the molecular structure and drug-sensitivity pattern of conventional LTCCs in electrically excitable cells. A common feature of these channels is their sensitivity to dihydropyridine (DHP) derivatives, such as nifedipine. The DHP receptor is well known originally as the α_1_-subunit of LTCCs in excitable cells [45]. LTCCs in neurons and myocytes are heterotetrameric polypeptide complexes consisting of a channel-forming α_1_-subunit, and at least three auxiliary subunits (α_2_/δ, γ and β) that specifically modulate the activity and allow depolarization-induced Ca^2+^ influx into the cytosol [45]. The predicted topology of the α_1_-subunit contains four repeated motifs (I–IV) and an inward-dipping loop between the S5 and S6 transmembrane segments that forms the channel pore, while the S4 transmembrane segment contains conserved positively charged amino acids that are voltage sensors and move outward upon membrane depolarization and open the Ca^2+^ channel by analogy with the voltage-gated K^+^ channel [46]. The spectrum of DHP derivatives, which specifically bind with high affinities to the α_1_-subunits of LTCCs and regulate their functional state from closed to open or vice versa, allows both the identification and functional analyses of this class of molecules. Human and rodent T cells express transcripts and/or proteins of the α_1S_ (Ca_v_1.1), α_1C_ (Ca_v_1.2), α_1D _ (Ca_v_1.3) and/or α_1F_ (Ca_v_1.4) subunits [47,48]. In addition, various splicing variants and isoforms of Ca_v_1.2, Ca_v_1.3 and Ca_v_1.4, together with auxiliary β-subunits, have been detected in human and mouse lymphocytes [47,48,49]. However, the issue of whether these channels are voltage-gated (gated by membrane depolarization) remains a matter of debate. It has been shown that LTCC agonists such as BayK8644 evoke robust Ca^2+^ influxes in Jurkat T cells and human peripheral blood T cells, which are blocked by the LTCC antagonist nifedipine [47]. On the other hand, in most experiments, high K^+^ loading alone evokes minimal Ca^2+^ influxes in these cells [48]. It should be noted that some variants lack the voltage-sensing S4 transmembrane segment [49], which may explain why the activation of LTCC-like channels is independent of membrane depolarization. Mast cells express Ca_v_1.2 and Ca_v_1. 3 and the LTCC activity is activated by antigen stimulation to regulate mediator release in a distinct manner from CRAC channels [50]. The lower expression of Ca_v_1.4 can only be observed by nested PCR. Similar to the conventional LTCCs in excitable cells and T cells, the LTCC activity observed in mast cells is activated independently of Ca^2+^ store emptying and is sensitive to DHP derivatives and other Ca^2+^ channel blockers. We recently reported that, similar to the case for antigen stimulation [51], high K^+^ loading evokes a robust Ca^2+^ influx in mast cells [50] that have been depleted of the ER Ca^2+^ stores, although thapsigargin induces no Ca^2+^ influx in these cells [51]. Moreover, both K^+^ and antigen stimulation induce substantial Ca^2+^ influxes into mitochondria in unmanipulated cells, and these Ca^2+^ responses are blocked by nifedipine, diltiazem and verapamil [50,51] or by gene knockdown of Ca_v_1.2 (unpublished data). Collectively, these observations suggest that certain LTCCs such as Ca_v_1.2 are activated by membrane depolarization and contribute to Ca^2+^ influx into mast cells. Thus, an emerging view is that LTCCs comprise alternative Ca^2+^ entry pathways in immune cells. Specifically, aspirin at low concentrations (≤0.3 mM) augments the LTCC activity, whereas at higher concentrations (>1 mM), it suppresses the LTCC activity [11]. More recently, we found that in mast cells with knocked down of Ca_v_1.2 gene expression, aspirin failed to affect the LTCC activity as well as Ca^2+^ influx, thereby indicating that Ca_v_1.2 mediates the effects of aspirin (unpublished data). Despite the essential role of external Ca^2+^ entry in generating LTC_4_ secretion, attention has only recently been paid to the Ca^2+^ channels involved in this entry. Biochemical analyses revealed that CRAC channels play key roles in AA release, cPLA_2_ activation and LTC_4_ secretion [52,53]. Recently, it has been revealed that the mammalian proteins stromal interaction molecule 1 (STIM1) and Orai1/CRAC modulator 1 (CRACM1) mediate the functions of CRAC channels (for reviews, see [54,55]). STIM1 senses the Ca^2+^ concentration in the ER and activates CRAC channels, while Orai1 is the pore-forming subunit of CRAC channels. The discovery of these molecules has enabled genetic analyses of the role of CRAC channels in LTC_4_ secretion in mast cells. It was revealed that LT secretion is strongly inhibited in mast cells derived from Orai1-knockout mice [56]. Thus, CRAC channels seem to be the major routes of Ca^2+^ entry involved in LTC_4_ secretion. Our data are apparently inconsistent with that view, since aspirin impairs CRAC channel activity but facilitates Ca^2+^ influx and LTC_4_ secretion. We found that even when CRAC channel activity is impaired, antigen stimulation still evokes robust LTC_4_ secretion and that aspirin augments this secretion [11]. Taken together with the aspirin-mediated facilitation of LTCC activity, these data support the view that an LTCC-mediated, CRAC channel-independent LTC_4_ secretion pathway exists, and that aspirin (and possibly salicylates) targets this pathway (Figure 1 and Figure 2). 

Although further studies are necessary to establish this view and the biological significance of such an alternative pathway, it should be noted that the LTCC-mediated LTC_4_ secretion pathway is facilitated by mitochondrial depolarization, which strongly impairs the CRAC channel-mediated Ca^2+^ influx and LTC_4_ secretion [11,52,57]. In the inflammatory milieu, mast cells may be exposed to oxidative stress, the major cause of mitochondrial depolarization, leading to inactivation of CRAC channel-mediated LTC_4_ secretion. It is likely that under such conditions, low doses of aspirin facilitate LTC_4_ secretion through the LTCC pathway, thereby leading to the exacerbation of allergic reactions, while high doses of aspirin block both of the two Ca^2+^ channel pathways, thereby strongly dampening LTC_4_ secretion (Figure 1). This scenario is consistent with the clinical observations that aspirin intolerance is induced by low doses of aspirin and that patients with aspirin intolerance can be desensitized to aspirin by oral challenges with high doses of aspirin, which results in reduced LT secretion [37,58,59,60]. Thus, unveiling the molecular mechanisms underlying NSAID modulation of Ca^2+^ channel activities could contribute to better understanding of their anti-inflammatory actions as well as their immunological side effects. 

## 4. Roles of ROS, Ca^2+^ and Mitochondria in the Chemopreventive Effects of NSAIDs

Much attention has been paid to the antineoplastic and chemopreventive effects of NSAIDs. Some clinical observations and epidemiological studies on numerous populations have revealed that prolonged use of aspirin and other NSAIDs reduces the risks of cancers of the colon and other gastrointestinal organs as well as those of the breast, prostate, lung and skin [13,14,15,16,17,18,19]. By definition, cancer chemoprevention is slowing, reversing or inhibiting carcinogenesis by the use of chemical agents, thereby lowering the risk of developing cancer. A growing list of agents including NSAIDs have been reported to have cancer chemopreventive activities, and many of them behave as apoptosis-inducing agents in animal and human cancer cells (for reviews, see [3,26,61,62,63]), consistent with the view that the commitment of these cells to cell death is an important mechanism underlying the chemopreventive effects. Different COX-independent mechanisms have been proposed to be involved in the chemopreventive and/or apoptosis-inducing effects of NSAIDs [3,26]. These mechanisms involve downregulation of NF-κB activity [8,64], inhibition of the protein kinase B/Akt pathway [65,66], alterations in the levels of proapoptotic- and antiapoptotic proteins [67,68,69], activation of extrinsic and intrinsic pathways of apoptosis [70,71,72,73] and modulation of glucose and energy metabolisms [74,75]. Among these, we focus on the activation of the intrinsic or mitochondrial apoptosis pathway, since the vast majority of putative chemopreventive agents, including retinoids (e.g., all-*trans* retinoic acid, 9-*cis*-retinoic acid, *N*-(4-hydroxyphenyl)retinamide), vanilloids (e.g., capsaicin and resiniferatoxin), rotenoids (rotenone and deguelin) and polyphenols (curcumin, epigallocatechin gallate and resveratrol) appear to initiate apoptosis via this pathway [61,63,64]. Besides their well-known role as the power plants in eukaryotic cells, mitochondria are now recognized as central gateway controllers of the intrinsic or mitochondrial apoptotic pathway. Permeabilization of the outer mitochondrial membrane (OMM) by proapoptotic Bcl-2 family proteins promotes the release of a number of apoptogenic factors, such as cytochrome c, endonuclease G, second mitochondrial activator of caspases, Omi/HtrA2 and apoptosis-inducing factor (AIF), from the inner mitochondrial membrane (IMM) space into the cytosol, and these apoptogenic proteins promote the activation of the caspase cascade, thereby leading to apoptosis. Cytochrome c interacts with the apoptotic peptidase activating factor 1, leading to the formation of the multimeric apoptosome in the presence of ATP/dATP [76,77]. 

The apoptosome then activates the initiator caspase (caspase 9), which subsequently cleaves and activates the effector caspases (caspases 3 and 7). A cytochrome c-independent apoptosis pathway has also been defined, and this pathway requires proteins such as endonuclease G and AIF to carry out apoptosis. Hence, in this paradigm, mitochondrial integrity disruption and downstream apoptogenic protein release and caspase activation play pivotal roles. Although the molecular mechanisms underlying the OMM permeabilization are poorly understood, there is general agreement in the literature that the mitochondrial permeability transition (MPT), which was originally defined as a sudden increase in the IMM permeability to solutes with molecular masses of ~1500 Da, is involved. It is now believed that opening of a putative megachannel referred as to the mitochondrial permeability transition complex (PTPC) occurs [78,79]. The PTPC is a high-conductance non-specific pore in the IMM that is composed of proteins that link the IMM and OMM. Several mitochondrial proteins localized in the IMM and OMM, such as voltage-dependent anion channels (VDACs), adenine nucleotide translocase (ANT), hexokinase, peripheral benzodiazepine receptors and cyclophilin-D are thought to constitute the PTPC. Under physiological conditions, the proteins in the OMM and IMM that constitute the PTPC are believed in close proximity to one another and in a closed or low conductance formation, although the PTPC has not been isolated and the components of this complex remain controversial [78,79,80]. When the PTPC changes to an open conformation, water and solutes with molecular masses of up to 1500 Da enter into the mitochondrial matrix, resulting in osmotic swelling of the mitochondrion. It has been believed that when multiple PTPCs open concurrently and extensive mitochondrial swelling takes place, physical disorganization of the OMM occurs and mitochondrial apoptogenic proteins are released, thereby triggering apoptosis [81]. Therefore, much attention has been paid to the potential role of PTPCs as a target for anticancer chemopreventive agents including NSAIDs [26,81,82]. For several reasons, reactive oxygen species (ROS) are believed to play a key role in MPT induction by affecting the PTPC conformation. First, ROS are byproducts of oxidative phosphorylation and excessive ROS generation is potentially deleterious to mitochondrial and cellular functions. Second, ANT has three cysteine residues whose oxidation is critical for PTPC open-closed transitions and Ca^2+^ release from the mitochondrial matrix, and PTPCs are believed to be particularly vulnerable to ROS [78,79,80]. Consequently, the MPT can be triggered by excessive mitochondrial ROS generation and/or disruption of the mitochondrial redox homeostasis [83,84,85]. Third, within mitochondria, cytochrome c is bound to the outer surface of the IMM by its association with the mitochondrial phospholipid cardiolipin, and oxidation of cardiolipin is thought to decrease this contact [86]. Thus, oxidation of cardiolipin may also be required to liberate sufficient cytochrome c to trigger caspase activation and induce apoptosis. The MPT also results in dissipation of the mitochondrial membrane potential and enhances ROS production via disintegration of the electron transport chain, thereby progressively shutting down oxidative phosphorylation and impairing energetic metabolism [87]. Hence, the MPT is a rate-limiting and self-amplifying process for apoptosis in which ROS play key roles. 

Another biochemical change that has been associated with the induction of apoptosis in several cell types is deregulation of the intracellular Ca^2+^ concentrations. Excessive intracellular Ca^2+^ levels, such as those induced by Ca^2+^ ionophores have been shown to induce apoptosis [88,89]. Moreover, apoptosis appears to involve a Ca^2+^-dependent endonuclease [90], and intracellular Ca^2+^ increases have been linked to apoptosis of both activated T cell hybridomas [91] and immature thymocytes [92]. In addition to its pro-apoptotic effects, Ca^2+^ has also been shown to act as an anti-apoptotic factor. IL-3-dependent primary cultured mast cells and cell lines can be protected against growth factor withdrawal-mediated apoptosis by the addition of Ca^2+^ ionophores [93], and programmed neuronal death is also suppressed by an increase in intracellular Ca^2+^ [94]. Collectively, Ca^2+^ appears to be necessary for both inducing and protecting against cell death, and the roles of Ca^2+^ in regulating cell death therefore seems to be more complex than initially thought. There is no general model that can depict the dual effects of Ca^2+^. It is now widely accepted that mitochondria play a key role in regulating intracellular Ca^2+^ concentrations. It is quite likely that an appropriate elevation in the mitochondrial Ca^2+^ concentration ([Ca^2+^]_m_) supports energy metabolism, cell activation and cell survival, whereas [Ca^2+^]_m_ overload causes increased cell death [95,96]. There is general agreement in the literature that [Ca^2+^]_m_ overload can damage mitochondrial integrity, thereby inducing PTPC opening [97,98] and resulting in the release of apoptogenic proteins. On the other hand, it has been shown that maintenance of [Ca^2+^]_m_ homeostasis is essential for cell survival, and that loss of [Ca^2+^]_m_ is closely correlated with cell death in cultured cells [99]. Collectively, ROS and Ca^2+^ are excellent targets for NSAIDs in regulating mitochondrial cell death. In fact, certain NSAIDs including aspirin, salicylates and aspirin analogs such as phosphoaspirin and nitric oxide (NO)-generating aspirin have been shown to exert proapoptotic effects on cancer cells via oxidative stress and/or ROS/NO generation [100,101,102]. However, it remains unclear whether the effects of NSAIDs on ROS generation are direct or indirect, and the molecular mechanisms of the oxidative responses are poorly understood. 

**Figure 3:**
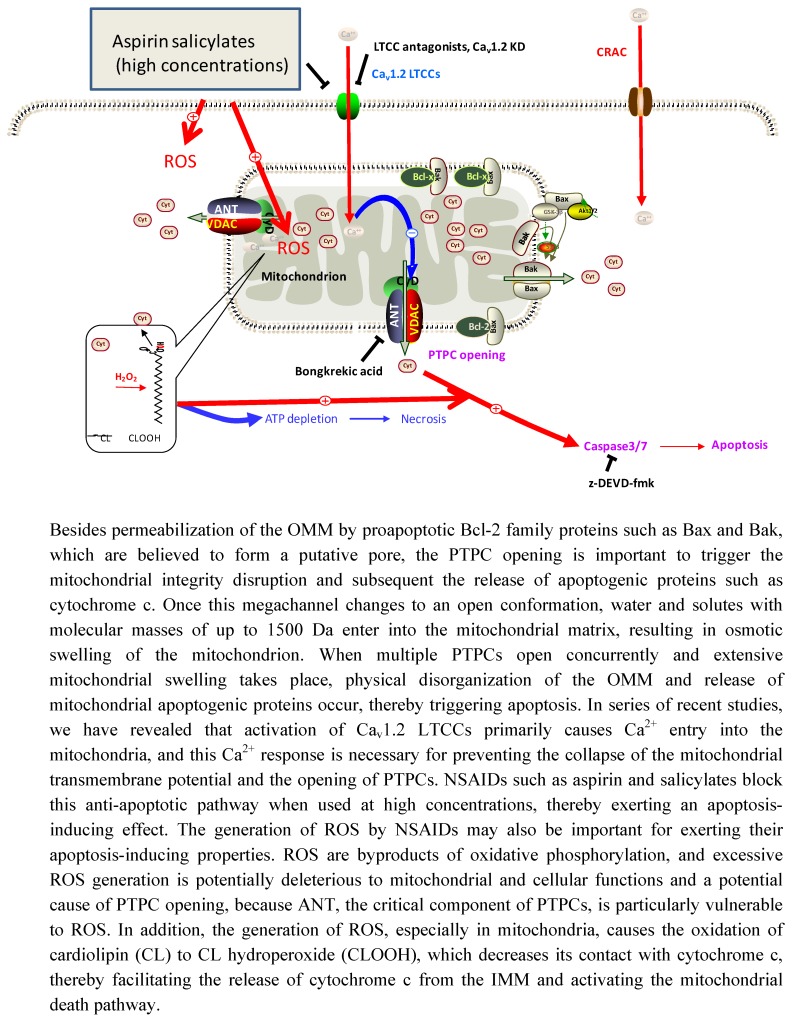
Proposed model for the apoptosis-inducing effects of NSAIDs.

There is much less available information regarding the effects of NSAIDs on cellular and mitochondrial Ca^2+^ concentrations. As mentioned above (Section 3), we recently found that aspirin modulates both CRAC channel and Ca_v_1.2 LTCC activities. One of the most attractive properties of Ca_v_1.2 LTCCs is their anti-apoptotic function. Ca_v_1.2 LTCCs protect mast cells against activation-induced cell death by preventing mitochondrial integrity collapse and the mitochondrial cell death pathway [103]. Pharmacological (e.g., LTCC antagonists) or genetic (gene knockdown) blockade of Ca_v_1.2 LTCC activity causes substantial apoptosis in activated cells. Moreover, activation (K^+^ loading) or augmentation (e.g., LTCC agonists) of Ca_v_1.2 LTCC activity protects mast cells against thapsigargin-induced apoptosis [103]. This prevention is accompanied by significant maintenance of the [Ca^2+^]_m_ levels (unpublished data). Taken together with our data that Ca_v_1.2 LTCCs are necessary for mitochondrial Ca^2+^uptake, it is quite possible that Ca^2+^ introduced via Ca_v_1.2 LTCCs is important for the maintenance of [Ca^2+^]_m_, thereby conveying a pro-survival signal. Consequently, blockade of LTCC-mediated anti-apoptotic Ca^2+^ signaling by relatively high concentrations of aspirin and salicylates may be a novel mechanism underlying their apoptosis-inducing effects (Figure 3). Specifically, we found that inhibition of Ca_v_1.2 LTCC activity affects the survival of tumor mast cells more markedly than that of primary mast cells [103], thereby suggesting that tumor cells rely more heavily on the LTCC-mediated pro-survival pathway than normal cells. In this regard, it should be noted that LTCC expression is up-regulated and/or LTCC activity is elevated in human cancer cells such as colon cancer and leukemia cells compared with their normal counterparts [104,105,106]. Moreover, the flavonoid wogonin has been shown to kill malignant T cells (in T cell leukemia), but not peripheral blood T cells by affecting LTCCs [106]. These observations are consistent with the view that cancer cells are more sensitive to the interference of LTCC activity than normal cells. We previously reported that NO generation via NO synthase (NOS) activity is necessary for the maintenance of cell mitochondrial integrity and cell survival in rat basophilic leukemia cells [107].

Subsequent studies revealed that endothelial NOS (eNOS) is essential for the generation of NO and activation of Ca_v_1.2 LTCCs [108]. Importantly, knockdown of the expression of Ca_v_1.2 LTCC [103] or eNOS [108] has minimal effects on cell survival in the resting state, thereby indicating that eNOS and Ca_v_1.2 LTCCs are specifically required for the survival of activated cells. Given that eNOS is activated by the PI3K-Akt pathway [107], it is most likely that NO generated by the PI3K-Akt-dependent eNOS activation pathway positively regulates the Ca_v_1.2 LTCC activity. Interestingly, the PI3K-Akt pathway and/or eNOS have been shown to play key roles in the survival of various cell types as well as in chronic inflammation and cancer [109,110,111,112,113,114,115]. Since the absence of Ca_v_1.2 LTCCs is significantly compensated for by blocking PTPC opening or inhibiting the downstream caspase cascade pathway, this type of Ca^2+^ channel may prevent extensive PTPC opening, thereby playing a key role in the maintenance of mitochondrial integrity. Taken together with several of the above-mentioned lines of evidence that (i) gene expression of LTCCs is up-regulated in cancer cells and LTCC activities are elevated compared with normal cells, (ii) cancer cell survival seems to rely more heavily on this type of Ca^2+^ channel than normal cell survival, (iii) these Ca^2+^ channel activities are necessary for the maintenance of mitochondrial integrity and prevention of apoptosis and (iv) several chemopreventive agents such as aspirin, salicylates and wogonin commonly affect these Ca^2+^ channel activities, LTCCs may be promising target molecules for cancer prevention and therapy. 

## 5. Conclusions and Perspectives

Recent studies have revealed unequivocally that certain NSAIDs exert their anti-inflammatory and cancer chemopreventive effects, as well as certain side effects, independently of COX activity and PG synthesis inhibition. It is very clear in the literature that multiple pathways are involved in these effects, but they are not shared by all NSAIDs. In this review, we have discussed the molecular basis of an emerging view that Ca^2+^ and mitochondria are novel and potentially more generalized targets for the biological effects of NSAIDs, as well as their side effects. If induction of apoptosis is the final goal of cancer chemopreventive drugs, better understanding of the molecular mechanisms underlying the aspirin-mediated modulation of PTPCs and LTCCs may help toward the development of cancer-selective drugs and/or therapies, since cancer cells seem to more sensitive to the modulation of these two types of channels than normal cells. 
